# Lower extremity energy absorption strategies at different phases during single and double-leg landings with knee valgus in pubertal female athletes

**DOI:** 10.1038/s41598-021-96919-y

**Published:** 2021-09-01

**Authors:** Mahdis Dadfar, M. Soltani, Mohammadreza Basohbat Novinzad, Kaamran Raahemifar

**Affiliations:** 1 Department of Corrective Exercise and Sports Injuries, Faculty of Physical Education and Sport Sciences, Allameh Tabataba’i University, Tehran, Iran; 2 Department of Mechanical Engineering, K. N. Toosi University of Technology, Tehran, Iran; 3 Department of Electrical and Computer Engineering, University of Waterloo, Waterloo, ON Canada; 4 Center for Biotechnology and Bioengineering (CBB), University of Waterloo, Waterloo, ON Canada; 5 Advanced Bioengineering Initiative Center, Computational Medicine Center, K. N. Toosi University of Technology, Tehran, Iran; 6 Cancer Biology Research Center, Cancer Institute of Iran, Tehran University of Medical Sciences, Tehran, Iran; 7 College of Information Sciences and Technology (IST), Data Science and Artificial Intelligence Program, Penn State University, State College, Pennsylvania, PA 16801 USA; 8 School of Optometry and Vision Science, Faculty of Science, Department of Chemical Engineering, Faculty of Engineering, University of Waterloo, 200 University Ave W, Waterloo, ON N2L 3G1 Canada

**Keywords:** Health care, Health occupations

## Abstract

Dynamic knee valgus (DKV) malalignment affects the biomechanical characteristic during sports activities. This cross-sectional study was conducted to evaluate mechanical energy absorption (MEA) strategies at initial contact (IC) and total landing (TL) phases during single-leg landing (SLL), and double-leg landing (DLL). Twenty-eight female athletes with DKV (age 10–14) were invited. MEA analysis of lower extremity joints was done in sagittal and frontal motion planes employing 8 Vicon motion capture cameras and 2 Kistler force plates. Statistical analysis was done using IBM Statistics (version24) by Bivariate Pearson Correlation Coefficient test. Knee extensors MEA during SLL (IC: P = 0.008, R = 0.522/TL: P < 0.001, R = 0.642) and DLL (IC: P < 0.001, R = 0.611/TL: P = 0.011, R = 0.525), and knee abductors during SLL (IC: P = 0.021, R = 0.474) were positively correlated with increased DKV angle. Ankle plantar flexors during SLL (TL: P = 0.017, R = − 0.477) and DLL (TL: P = 0.028, R = − 0.404), and hip extensors during SLL (TL: P = 0.006, R = − 0.5120) were negatively correlated with increased DKV angle. Compensated MEA in knee extensors was correlated with less ankle plantar flexion MEA during SLL (IC: P = 0.027, R = − 0.514/TL: P = 0.007, R = − 0.637) and DLL (IC: P = 0.033, R = − 00.412/TL: P = 0.025, R = − 0.485). These outcomes indicated a knee-reliant MEA strategy in female athletes with DKV during puberty, putting them at higher risks of ACL injuries during landing.

## Introduction

Dynamic knee valgus (DKV) is known as a malalignment involving the lower extremity joints such as excessive abduction and or internal rotation in the knee joint, excessive adduction and internal rotation in the hip joint, limited range of ankle dorsiflexion, and foot pronation, more commonly observed in female athletes during sports activities^1–4^. Female athletes are reported to incline to develop greater DKV during the initial contact (IC) phase of a landing task, putting them at higher risks of Anterior Cruciate Ligament (ACL) injury occurrence compared to their male counterparts^4^. Female athletes are also reported to exhibit greater knee valgus during the pubertal growth, induced as a consequence of insufficient neuromuscular adaptations to the skeletal system growing rapidly^5^. Based on a longitudinal biomechanical analysis, female athletes are more prone to experience increased peak abduction angle and moment in the knee joint following the pubertal growth^6^. Given the fact that 70% of ACL injuries are non-contact, abnormal biomechanical profiles such as excessive knee abduction could play a crucial role in increasing ACL strain^2,7,8^. Biomechanical findings indicated that ACL non-contact injuries increasing with the higher rate of sports participation could occur more prevalently in female athletes following puberty than adult females^9^. While the number of young athletes participating in competitive sports increases between 11 and 13 years of age^10^, sustaining ACL injury may put an end to their athletic career at the early stages and hinder teams in-season performance^11^. Additionally, knee joint injuries in younger ages may reduce participation in recreational physical activities, increasing many health-related problems in adulthood^12,13^. Moreover, ACL strain may decrease the injured athlete’s quality of life in the long-term by triggering meniscus injuries ^14^, developing early onset knee osteoarthritis between the ages from 30 to 50^15^, and total knee replacement as they turn to adulthood^16^.

Regarded as high-risk tasks for ACL injuries, single and double leg landings are reportedly one of the most repeated movements during many sports, especially in volleyball and basketball^2^. The imbalanced loading patterns and impact stress imposed on the knee joint during landing tasks in the mentioned sports have been reported as the reasons behind the greater risk of non-contact ACL injury compared to other physical activities^2,17^. Recent findings, however, showed different biomechanical characteristics during unilateral or bilateral landing. While athletes tend to land with greater hip and knee flexion angle during double-leg landing (DLL) compared to single-leg landing (SLL)^18^, greater knee abduction moment was observed during SLL compared to DLL^19^. Additionally, a previous study reported differences in mechanical energy absorption (MEA) strategy when comparing SLL and DLL tasks in healthy adult males. Based on their results, the major contributors to absorb mechanical energy during DLL were hip (at sagittal and frontal planes) and knee (at sagittal plane) joints, while knee (at sagittal and frontal planes) and ankle joints (at sagittal plane) had greater contribution during SLL task^20^. A previous study also indicated that athletes might adopt different MEA strategies during SLL and DLL tasks^20^. This shows the importance of evaluating kinematic characteristics in addition to giving the predictability of injurious MEA strategies during landing to design a practical injury prevention exercise program for female athletes during puberty^21^. However, there is not enough evidence on evaluating MEA during different phases of landing tasks in female athletes developing knee valgus during the pubertal growth.

Developing MEA strategies is reported to be different between genders and age groups, as knee extensors showed to have greater contribution to absorb mechanical energy in females going through pubertal growth spurt to post-pubertal years when the highest rate was observed, compared to younger or adult female or males^22^. Biomechanical analysis of female athletes landing pattern indicated that they have a tendency to a knee dominant MEA strategy compared to their male counterparts^23^. The plausible reasons behind this sex-related difference are reported to be related to higher quadriceps muscles engagement compared to hamstring muscles, greater knee valgus collapse, and more erect landing pattern observed in females^23–25^. In a recent study, female athletes with knee valgus showed greater MEA of knee extensors during landing compared to the ones without valgus posture^26^. Previous findings have also indicated different adopted MEA strategies when comparing lower extremity energetics between IC and total landing (TL) phases during the deceleration phase of jump-landing^27^. This phase-related difference is caused on account of the differences in magnitudes of ground reaction forces (GRF) and joints angular displacements between IC and TL phases during landing^27–29^. Based on the biomechanical evidence, greater MEA in the sagittal motion plane observed during the IC phase may be produced due to higher extension moment and posterior GRF on the knee joint at the initial phase of landing^30^. Likewise, the observed knee-dominant MEA strategy in the frontal motion plane at the IC phase could be linked with greater knee valgus and hip adduction angles at the initial landing^31^. Therefore, comparing MEA strategies during different phases of landing tasks would provide profound insight into injurious biomechanical profiles for clinicians and coaches.

Thus, the purpose of the current study was to compare lower extremity MEA strategies in the sagittal and frontal motion planes at IC and TL phases of SLL and DLL tasks in female participants exhibiting DKV during puberty.

## Methods

### Study design and participants

Twenty-eight female athletes during puberty (10–14 years of age) with a history of regular sport activity in volleyball and basketball were invited for this cross-sectional study. Participants were determined to have DKV using single-leg-squat (SLS) test initially. In doing so, participants with at least 2 out of 3 repetitions with noticeable or significant valgus in both legs during SLS were recruited (Noticeable valgus: when patella was pointed towards the second toe, Significant valgus: when patella was pointed past inside the second toe)^32,33^. In the secondary assessment session, the knee valgus angle was recorded by 3-D motion analysis cameras during SLL and DLL landing tasks in the laboratory setting. Participants with knee valgus angle greater than 4.4° ± 3.0° at TL during both landings were selected for this study^34^. The exclusion of this study included: any pain in the spine or lower extremity, and history of musculoskeletal injuries in the last year or fracture in the lower extremity, cardiovascular diseases, balance impairments, undergoing surgery for spine or lower extremity, using any medicine, drug, or painkiller during the time of the study, and observing any musculoskeletal malalignment based on the New York test. Additionally, participants who could not complete the landing tests due to any pain, discomfort, or musculoskeletal disorders were excluded from the study. The current study was approved in terms of ethical considerations by the Committee for Ethics in Biomedical Research of the University of Social Welfare and Rehabilitation Sciences after obtaining the ethics code (IR.USWR.REC.1398.007), and all methods were performed in accordance with relevant guidelines and regulations. Since participants of this study were under the legal age, the informed consent was obtained from a parent and/or legal guardian of all the participants. The obtained data for this study were promised to stay confidentially between the researchers only. The demographic characteristics of the participants are provided in (Table 1).

**Table 1:** Demographic characteristics and determined DKV angle (SLS test: initial selection, 3-D analysis during SLL/DLL: secondary selection).

Variables	Mean ± SD (N = 28)
Age	12.41 ± 2.04
Wight	45.26 ± 6.32
Height	1.55 ± 8.01
BMI	19.71 ± 2.3
SLS test noticeable valgus score	2.62 ± 0.82
SLS test significant valgus score	1.71 ± 0.34
SLL 3-D analysis DKV angle°	− 9.35° ± 3.74°
DLL 3-D analysis DKV angle°	− 5.64° ± 3.28°

### Instrumentation

**Figure 1:**
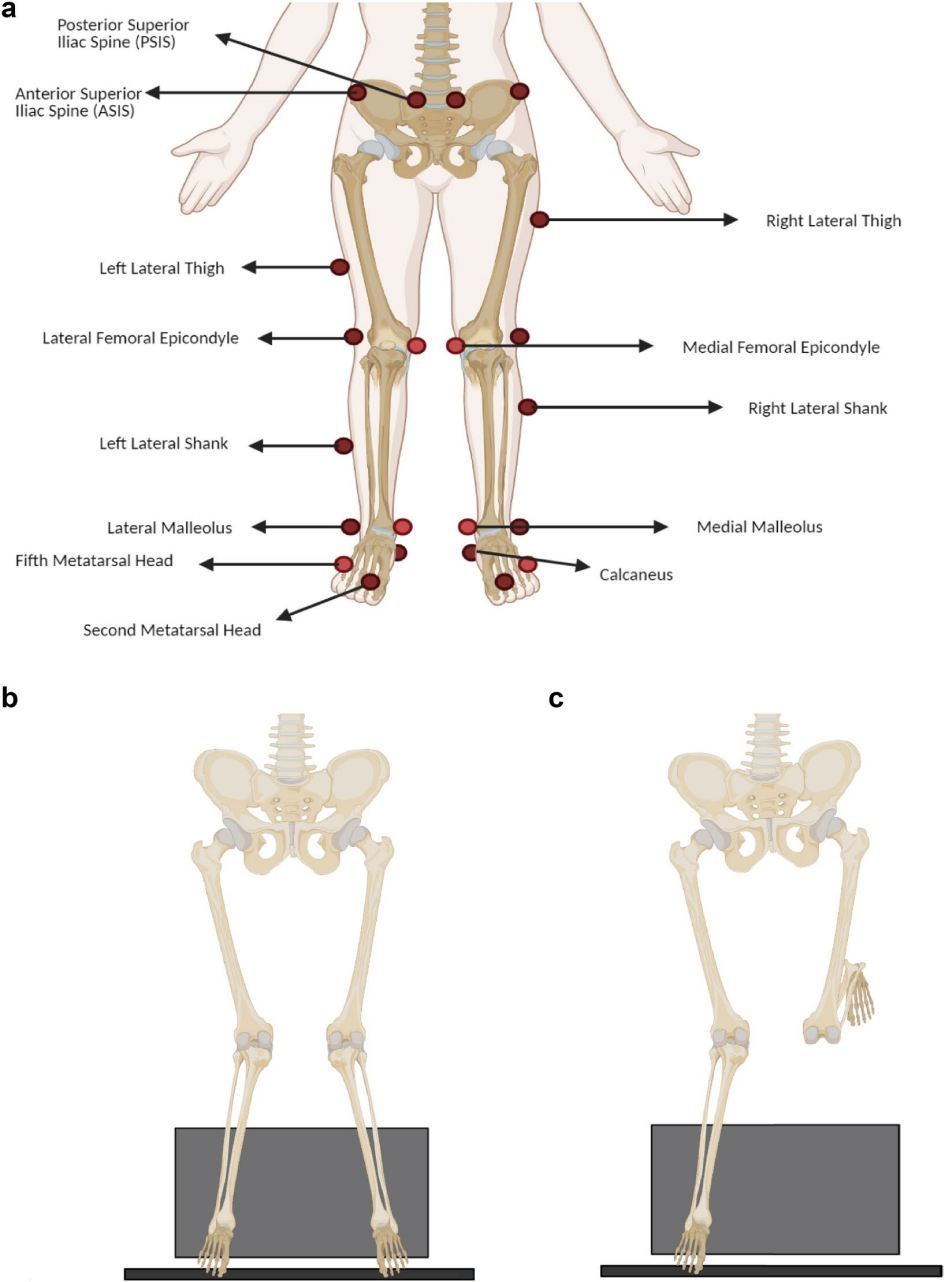
Lower extremity marker placement: 20 retroreflective markers based on the modified plug-in-gait marker system/B: DLL task, and C: SLL task from a box (30 cm height from the floor).

All of the testing procedures of the current study were completed in the Laboratory of Movement Disorders, Department of Physical Therapy, at the University of Tarbiat Modares. For analyzing kinematics and kinetics during both landing tasks, eight Vicon motion capture cameras (120 Hz—2.2 mega-pixel Vero model cameras—UK), and two synched Kistler force plates (1200 Hz—model 9286ba—40 cm × 60 cm dimension—Switzerland) embedded in-floor were used. During the testing procedure, motion capture cameras and force plates were calibrated based on the Vicon system manufacturing recommendation. Data collection and processing of the raw data were done based on the recommended plug-in-gait method by Vicon and Nexus software (version 2.9). Twenty-two retroreflective markers were placed on lower extremity landmarks based on the Plug-in-Gait marker system: laterally on posterior superior iliac spine (PSIS), anterior superior iliac spine (ASIS), lateral thigh, lateral femoral epicondyle, lateral shank, lateral malleolus, second metatarsal head, and calcaneus. Additional markers were placed on the fifth metatarsal, medial malleolus and medial femoral epicondyle, which were used for anthropometric assessments^35^ (Fig. 1).

### Experimental procedure

#### DKV angle determination by SLS test

Single-leg-squat test was employed to initially determined DKV in participants. Before starting the assessment procedure, the examiner performed and explained a correct SLS to the participants. During the test, participants were asked to fold their arms on the chest and perform SLS with the knee flexion between 45° to 60° while keeping the other leg flexed from the hip with straight knees. SLS evaluation was qualitative in the current study, and participants were scored on the SLS paper sheet^33^. A trial was considered acceptable if these criteria were conformed: to keep hands on the chest during the whole procedure, to reach the desired knee flexion, and to keep the squat position at least for 5 s. This test was repeated three times with both dominant and non-dominant legs.

#### DKV angle determination by 3-D analysis

Dynamic knee valgus angle was recorded using 3-D analysis during SLL and DLL. During the assessment session, participants were asked to land from a 30 cm-box unilaterally on their dominant leg determined by a shooting questionnaire^36^. Knee valgus angle was determined using Euler angles from the relative orientations of the femur and tibia. This test was repeated thrice.

#### SLL test

Participants stood on the dominant leg on a 30 cm-box located 70 cm behind the center of the force plate. The non-dominant leg was flexed 90° backwards from the knee joint. During the whole test procedure, participants were asked to keep their hands on their waist, and jumped from the box by the examiner’s verbal order and landed on the same leg while keeping the other leg flexed from the knee joint. Participants were asked to keep their balance after landing for 5 s. This test was repeated thrice (Fig. 1).

#### DLL test

The test’s procedure was similar to the single-leg landing test, but participants landed on both feet. Criteria to collect a correct trial in both landing tests were: to hold the forward-looking posture without looking down on the force plate, to maintain the balance after landing for 5 s, to land on the center of the force plate and not the corners, not to perform the tests with arm swings or wobbly landing patterns, and not to perform jump-landings with an upward or forward jumping pattern. Both landing tests were repeated three times (Fig. 1).

### Data collection

**Figure 2:**
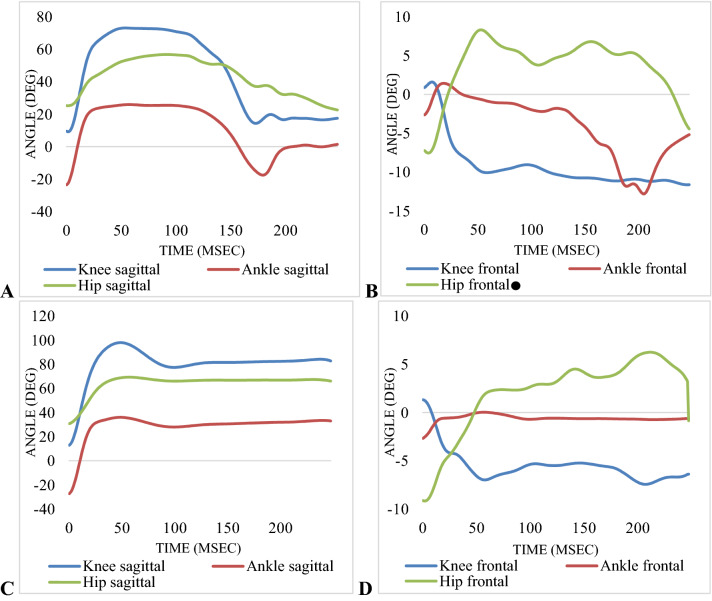
(**A**) Angle curves of knee, ankle, and hip joints from IC to TL phases: (**A**) in sagittal motion plane during SLL, (**B**) in frontal motion plane during SLL, (**C**) in sagittal motion plane during DLL, (**D**) in frontal motion plane during DLL. (+ values = flexion, dorsiflexion, adduction/− values = extension, plantar flexion, abduction)/ = Significant correlation observed between hip adduction angle and DKV angle during SLL (P = 0.043, R = 0.327).

Collecting and processing raw data were done by Vicon and Nexus software (version 2.9) based on the recommended plug-in-gait method. Woltring filter (MSE10) was used to smooth and filter data under 20 frames. Each joint angle in sagittal and frontal motion planes was obtained by calculated Euler angles (Fig. 2). DKV angle was derived from relative orientations of the distal segment (tibia) and proximal segment (femur)^26^. The extracted anthropometric data, which were assessed in the static position, were then applied to dynamic data. The primary measured outcomes of the current study included hip, knee, and ankle joints MEA in sagittal and frontal motion planes at IC and TL during SLL and DLL tasks calculated from integration of absolute values of the power-time curve^28^. Motions of each joint were defined separately for the hip joint as the thigh motions relative to the pelvis, for the knee joint as shank motions relative to the thigh, and for the ankle joint as foot motions relative to the shank^30^. Knee extensors MEA was defined as the amount of absorbed energy by eccentric contraction of quadriceps muscle during deceleration phase (landing phase), and hip extensors MEA was defined as the amount of absorbed energy by eccentric contraction of gluteus maximus during deceleration phase (landing phase)^31,37^. IC time point was defined as a moment when the vertical ground reaction force (VGRF) threshold exceeded 10 N during different tasks. TL time point was determined as the moment when each individual reached maximum knee flexion and kept this stable position for 5 s. Power-time curve values were calculated by recorded joints angular velocity, linear velocity, net joint moment, and forces (normalized to body weight/kg and height/m)^38^. The mechanical energy analysis was done using Python software (version 3.8).

### Statistics

Statistical analysis of the current study was done using IBM SPSS (version 24). Shapiro–Wilk's test was run to examine the data normality distribution. Bivariate Pearson Correlation Coefficient test was run to determine the correlations between MEA in each joint and DKV angle, and to identify any significant correlations between the compensatory MEA strategies of each joint at IC and TL phases during SLL and DLL tasks. P-values less than (α ≤ 0.05) were considered statistically significant. It is hypothesized that a knee-dominant MEA would be adopted during landing tasks by female athletes developing DKV posture during puberty.

### Ethical considerations

The current study was approved in terms of ethical considerations by the Committee for Ethics in Biomedical Research of the University of Social Welfare and Rehabilitation Sciences after obtaining ethics code (IR.USWR.REC.1398.007).

## Results

### MEA differences between IC and TL during SLL

**Figure 3:**
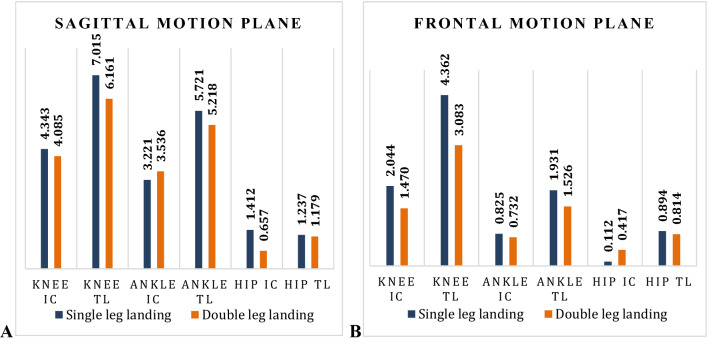
Means of each joint contribution to MEA (J/kg m) compared between different phases during SLL and DLL (IC: initial contact/TL: total landing). (**A**) Sagittal motion plane: knee extensors/ankle plantar flexors/hip extensors. (**B**) Frontal motion plane: knee abductors/ankle abductors/hip abductors.

Based on the statistics, knee extensors MEA (IC: P = 0.008, R = 0.522/TL: P < 0.001, R = 0.642) and knee abductors (IC: P = 0.021, R = 0.474) were positively correlated with DKV angle in female athletes during puberty. Additionally, MEA of hip extensors (TL: P = 0.006, R = − 0.5120) and ankle plantar flexors (TL; P = 0.017, R = − 0.477) were negatively correlated with increased DKV angle (Table 2, Fig. 3).

**Table 2:** Correlation between knee, ankle, and hip joints MEA (J/kg m) in sagittal and frontal motion planes at IC and TL phases during SLL and DLL and DKV angle during SLL (− 9.35° ± 3.74°) and DLL (− 5.64° ± 3.28°)/* = Significant variables/negative R-score = inverse correlation, positive R-score = positive correlation.

Variables	IC: Mean ± SD	IC: P-value	IC: R-score	TL: Mean ± SD	TL: P-value	TL: R-score
Knee extensors—SLL	4.343 ± 1.716	0.008*	0.522	7.015 ± 4.119	< 0.001*	0.642
Ankle plantar flexors—SLL	3.221 ± 0.368	0.418	0.019	5.721 ± 0.833	0.017*	− 0.477
Hip extensors—SLL	1.412 ± 0.080	0.226	0.112	1.237 ± 0.813	0.006*	− 0.512
Knee abductors—SLL	2.044 ± 1.557	0.021*	0.474	4.362 ± 1.016	0.118	0.285
Ankle abductors—SLL	0.825 ± 0.064	0.153	**− **0.237	1.931 ± 0.157	0.770	**− **0.009
Hip abductors—SLL	0.112 ± 0.311	0.59	**− **0.062	0.894 ± 0.022	0.518	0.042
Knee extensors—DLL	4.085 ± 0.191	< 0.001*	0.611	6.161 ± 3.420	0.011	0.525
Ankle plantar flexors—DLL	3.536 ± 1.769	0.388	0.091	5.218 ± 1.200	0.028*	− 0.404
Hip extensors—DLL	0.657 ± 0.033	0.404	0.016	1.179 ± 0.022	0.322	**− **0.185
Knee abductors—DLL	1.470 ± 0.044	0.613	**− **0.006	3.083 ± 0.071	0.089	0.303
Ankle abductors—DLL	0.732 ± 0.218	0.242	**− **0.255	1.526 ± 0.080	0.472	**− **0.011
Hip abductors—DLL	0.417 ± 0.332	0.322	**− **0.181	0.814 ± 0.022	0.083	**− **0.378

### MEA differences between IC and TL during DLL

Based on the statistics, knee extensors MEA (IC: P < 0.001, R = 0.611/TL: P = 0.011, R = 0.525) was positively correlated with increased DKV angle in female athletes during puberty. Additionally, MEA ankle plantar flexors (TL: P = 0.028, R = − 0.404) was negatively correlated with increased DKV angle (Table 2, Fig. 3).

### Compensated MEA during SLL

Statistical comparisons indicated MEA of ankle plantar flexors to be negatively correlated with MEA of knee extensors (IC: P = 0.027, R = –0.514/TL: P = 0.007, R = − 0.637) (Table 3).

**Table 3:** Compensated MEA (J/kg m) strategies relative to knee joint in sagittal and frontal motion planes at IC and TL during SLL and DLL/* = Significant variables/ negative R-score = inverse correlation, positive R-score = positive correlation.

Variables	IC: P-value	IC: R-score	TL: P-value	TL: R-score
Ankle plantar flexors vs knee extensors—SLL	0.027*	− 0.514	0.007*	− 0.637
Hip extensors vs knee extensors—SLL	0.672	**− **0.007	0.521	0.009
Ankle abductors vs knee abductors—SLL	0.216	0.145	0.067	0.402
Hip abductors vs knee abductors—SLL	0.513	0.013	0.332	0.211
Ankle plantar flexors vs knee extensors—DLL	0.033*	− 0.412	0.025*	0.485
Hip extensors vs knee extensors—DLL	0.366	0.026	0.232	**− **0.114
Ankle abductors vs knee abductors—DLL	0.19	**− **0.126	0.184	**− **0.214
Hip abductors vs knee abductors—DLL	0.857	**− **0.004	0.513	**− **0.017

### Compensated MEA during DLL

Statistical comparisons of MEA compensatory strategies during SLL in female athletes during puberty indicated MEA of ankle plantar flexors to be negatively correlated with MEA of knee extensors (IC: P = 0.033, R = − 0.412/TL: P = 0.025, R = − 0.485) (Table 3).

## Discussion

The purpose of the current study was to compare the compensatory MEA strategies at the IC and TL phases of SLL and DLL tasks in female athletes with DKV posture during puberty. Results of this study indicated a knee-dominant MEA strategy in sagittal motion plane at IC and TL phases during both landing tasks and in frontal motion plane at IC phase during SLL to be significantly correlated with increased DKV angle. Moreover, increased MEA of knee extensors showed to be the compensatory strategy to less MEA of ankle plantar flexors at IC and TL during both landing tasks.

In line with previous findings, results of this study showed greater knee extensors MEA at IC and TL during SLL and DLL tasks to be correlated with increased DKV angle^26,39^. Likewise, a previous study indicated greater knee extensors engagement to MEA during DLL task in female adults with valgus posture, compared to neutral or varus alignments^39^. This knee-dominant MEA strategy in the sagittal plane was reported to be caused due to female athletes’ greater tendency to rely on knee extensors (quadriceps muscle group) for initial shock attenuation during landing tasks^23,26,40^. Additionally, the changes in musculoskeletal and endocrine systems during the pubertal growth were associated with altered biomechanical characteristics observed in these ages^41,42^. Delayed muscular pre-activation observed in prepubescent boys was also suggested as another reason behind a different MEA observed in athletes during puberty compared to adults^41^. However, there is not enough evidence on evaluating muscular contraction patterns in female athletes during puberty, and further studies are needed on this matter. The imbalanced MEA strategies (higher knee extensors negative work compared to ankle and hip joints) during landing in girls following puberty can be considered to increase the stress imposed on the ACL^30^. Findings of the current study also indicated greater knee abductors contribution to MEA at IC phase during SLL, which was in contrast to a previous study showing a negative correlation between knee abductors negative work and DKV angle^26^. The main reason behind observing a knee-dominant MEA in the frontal plane at the IC phase of the SLL test relative to DKV malalignment might be the greater knee abduction moment, and valgus collapse observed during the initial phase SLL landing in participants with knee valgus^25^. Additionally, previous studies indicated that participants with DKV would be more prone to develop unbalanced neuromuscular control in the hip and knee joints which MEA strategies^23,43^. The results of the current study also showed smaller hip extensors MEA to be correlated with greater DKV angle only at the TL phase of the SLL task. Likewise, a previous study observed less contribution of hip extensors during side-stepping maneuvers in female adults exhibiting valgus posture^26^. There would be some reasons behind a smaller hip extensors contribution to MEA observed in the current study such as the decreased neuromuscular control and altered activation patterns in the muscles surrounding the hip joint observed in females compared to males^44,45^, and insufficient neuromuscular control in the hip and knee joints observed in females developing valgus posture compared to females without valgus posture^2,22,46^. Moreover, the inclination to a higher quadriceps activation pattern in females might be another causing factor for developing a knee-reliant strategy^47^. The reason behind the task-related difference in hip extensors MEA outcomes between SLL and DLL would be related to a quadriceps-dominant activation pattern during more physically challenging tasks such as SLL, in exchange of smaller hip extensors contribution to MEA^48,49^. However, a previous study observed less MEA of hip extensors during DLL task in adult females with valgus posture^39^. This difference in the outcomes related to the hip MEA between our findings and the previous one was assumed to rely on the different age group of the participants, since females between 10 and 15 years of age were observed adopt altered hip extensor moments compared to the ones above this age group^22^. Findings of this study also indicated smaller MEA of ankle plantar flexors to be significantly correlated with increased DKV angle at TL phase during both landing tasks, which was not observed at IC phase. Further, the results of this study were in line with previous findings reporting smaller mechanical work of ankle plantar flexors during the maximum phase of a side-stepping task in adult female athletes with valgus posture^26^. The key behind this phase-related difference might be the greater compensated MEA of knee extensors at TL phase compared to IC phase reducing the ankle plantar flexors role in MEA in female athletes with knee valgus^39^. Comparing male children basketball players MEA strategies to adults during the landing phase of a drop jump task indicated greater rates of peak impact forces at a shorter energy absorption phase, in addition to increased peak extensor moments at the lower extremity joints compared to adult males. However, their results did not show any difference in the MEA strategy between adolescent and adult males^41^. Additionally, comparing female and male athletes during a period from pre-pubertal years to young adulthood (9–22 years) indicated that knee/hip energy absorption ratio in female athletes started to increase from pubertal to post-pubertal years, with the highest ratio observed during post-pubertal. This ratio was reported to reach its highest levels among male and female athletes during other age groups^22^. However, there is not enough information regarding the MEA strategies relative to exhibited DKV angle in female athletes during puberty.

As hypothesized, outcomes of the current study indicated that increased MEA of knee extensors was the compensatory strategy developed in response to less MEA of ankle plantar flexors at IC and TL phases during SLL and DLL tasks. This finding was consistent with a previous study considering female athletes with DKV posture more inclined to knee joint injuries due to the imbalanced MEA in the sagittal motion plane^26^. Previous findings also showed children to have a greater tendency to increased knee extensors contribution to total MEA due to the insufficient co-contraction patterns observed in muscles around the knee joint such as hamstrings and vastus medialis^50^. Additionally, greater MEA of knee abductors was found to be caused on account of the excessive frontal plane loadings imposed on the knee joint at the IC phase, increasing the risk of valgus collapse, consequently^51^. However, no study has evaluated the compensatory strategies in adolescents or female athletes exhibiting DKV posture, and more previous ones were aimed at participants with knee osteoarthritis.

The observed compensated MEA in knee extensors relative to limited MEA of ankle plantar flexors during landing tasks was considered to be adopted in order to prevent the dynamic collapse of the joints and to maintain the lower extremity stability during jump landings in participant exhibiting knee valgus^52^. Based on the previous evidence, this knee-dominant MEA compensatory strategy was considered to increase the risks of ACL strains during physical activities by putting tremendous mechanical stress on the joint^39^. Additionally, the results of this study indicated knee abductors to have a higher contribution to MEA at the IC phase of the SLL task, putting excessive stress on the knee joint^51^. These differences become more important for females during puberty with greater inclination to participate in professional sports field^10^, exhibiting different neuromuscular strategies during physical activities^41^, higher sensitivity to musculoskeletal injuries especially ACL injuries at late pubertal growth^9,41^, and longer rehabilitation duration putting an end into athletic career^53^. Additionally, a previous study indicated that female athletes following the pubertal growth between 12 to 15 years of age were more susceptible to experience greater hip and knee extensor moments rations as a predictor of ACL injuries than male athletes at the same age^22,39^. Thus, when designing the injury prevention protocols, reaching a profound understanding of the compensated strategies during different phases of jump landing tasks would be useful for mitigating the injurious factors to the knee joint in female athletes demonstrating DKV during puberty compared to adults^39^. However, yet there is not enough evidence to confirm MEA strategies adopted by females during puberty who exhibit malalignment such as knee varus, or valgus and its differences to neutral posture in females during puberty or at different age groups.

The current study had two limitations. The first one is the inclusion of female athletes only. Since previous studies reported distinct biomechanical characteristics between male and female athletes, we cannot generalize the results of this study to male athletes. The second limitation of this study includes the age bracket aimed at females between 10 and 14 years old. Due to the reported age-related differences in the lower extremity biomechanics, the outcomes of this study cannot be generalized to the younger or elder age groups^41^.

## Conclusion

Results of this study showed greater knee extensors MEA to be correlated with increased DKV angle at IC and TL phases of SLL and DLL tasks indicating a knee-dominant MEA strategy in the sagittal motion plane in pubertal female athletes with DKV. Additionally, knee abductors showed to have a prominent role in adsorbing mechanical energy at the IC phase during the SLL representing higher valgus loadings. The observed compensated MEA in knee extensors during landings relative to smaller negative work of ankle plantar flexors and hip extensors may be the explanatory reason behind higher rates of knee joint loading and ACL strain during landing in female athletes developing DKV during puberty.

## References

[1] Zazulak B (2005). Gender comparison of hip muscle activity during single-leg landing. J. Orthop. Sports Phys. Ther..

[2] Hewett TE (2005). Biomechanical measures of neuromuscular control and valgus loading of the knee predict anterior cruciate ligament injury risk in female athletes: A prospective study. Am. J. Sports Med..

[3] Dugan SA (2005). Sports-related knee injuries in female athletes: What gives?. Am. J. Phys. Med. Rehabil..

[4] Russell KA, Palmieri RM, Zinder SM, Ingersoll CD (2006). Sex differences in valgus knee angle during a single-leg drop jump. J. Athl. Train..

[5] Hewett TE, Myer GD, Ford KR (2004). Decrease in neuromuscular control about the knee with maturation in female athletes. J. Bone Jt. Surg. Am. Vol..

[6] Ford KR, Shapiro R, Myer GD, Van Den Bogert AJ, Hewett TE (2010). Longitudinal sex differences during landing in knee abduction in young athletes. Med. Sci. Sports Exerc..

[7] Boden BP, Sheehan FT, Torg JS, Hewett TE (2010). Noncontact anterior cruciate ligament injuries: Mechanisms and risk factors. J. Am. Acad. Orthop. Surg..

[8] Agel J, Arendt EA, Bershadsky B (2005). Anterior cruciate ligament injury in national collegiate athletic association basketball and soccer: A 13-year review. Am. J. Sports Med..

[9] Sanders TL (2016). Incidence of anterior cruciate ligament tears and reconstruction: A 21-year population-based study. Am. J. Sports Med..

[10] Maffulli N (2000). At what age should a child begin regular continuous exercise at moderate or high intensity?. West J. Med..

[11] Tirabassi J (2016). Epidemiology of high school sports-related injuries resulting in medical disqualification: 2005–2006 through 2013–2014 academic years. Am. J. Sports Med..

[12] Maffulli N, Longo UG, Gougoulias N, Loppini M, Denaro V (2010). Long-term health outcomes of youth sports injuries. Br. J. Sports Med..

[13] Emery CA (2003). Risk factors for injury in child and adolescent sport: A systematic review of the literature. Clin. J. Sport Med..

[14] Kilcoyne KG, Dickens JF, Haniuk E, Cameron KL, Owens BD (2012). Epidemiology of meniscal injury associated with ACL tears in young athletes. Orthopedics.

[15] Friel NA, Chu CR (2013). The role of ACL injury in the development of posttraumatic knee osteoarthritis. Clin. Sports Med..

[16] Suter LG (2017). Projecting lifetime risk of symptomatic knee osteoarthritis and total knee replacement in individuals sustaining a complete anterior cruciate ligament tear in early adulthood. Arthritis Care Res..

[17] Herrington L (2011). Knee valgus angle during landing tasks in female volleyball and basketball players. J. Strength Cond. Res..

[18] Taylor JB, Ford KR, Nguyen A-D, Shultz SJ (2016). Biomechanical comparison of single-and double-leg jump landings in the sagittal and frontal plane. Orthop. J. Sports Med..

[19] Lim B-O, An K-O, Cho E-O, Lim S-T, Cho J-H (2021). Differences in anterior cruciate ligament injury risk factors between female dancers and female soccer players during single-and double-leg landing. Sci. Sports.

[20] Yeow CH, Lee PVS, Goh JCH (2011). An investigation of lower extremity energy dissipation strategies during single-leg and double-leg landing based on sagittal and frontal plane biomechanics. Hum. Mov. Sci..

[21] Rössler R (2014). Exercise-based injury prevention in child and adolescent sport: A systematic review and meta-analysis. Sports Med. (Auckland, NZ).

[22] Sigward S, Pollard C, Powers C (2012). The influence of sex and maturation on landing biomechanics: Implications for ACL injury. Scand. J. Med. Sci. Sports.

[23] Decker MJ, Torry MR, Wyland DJ, Sterett WI, Steadman JR (2003). Gender differences in lower extremity kinematics, kinetics and energy absorption during landing. Clin. Biomech. (Bristol, Avon).

[24] Cortes N (2007). Effects of gender and foot-landing techniques on lower extremity kinematics during drop-jump landings. J. Appl. Biomech..

[25] Myer GD, Ford KR, Hewett TE (2004). Rationale and clinical techniques for anterior cruciate ligament injury prevention among female athletes. J. Athl. Train..

[26] Tamura A, Akasaka K, Otsudo T (2020). Lower-extremity energy absorption during side-step maneuvers in females with knee valgus alignment. J. Sport Rehabil..

[27] Norcross MF, Blackburn JT, Goerger BM, Padua DA (2010). The association between lower extremity energy absorption and biomechanical factors related to anterior cruciate ligament injury. Clin. Biomech. (Bristol, Avon).

[28] Devita P, Skelly W (1992). Effect of landing stiffness on joint kinetics and energetics in the lower extremity. Med. Sci. Sports Exerc..

[29] Zhang S, Bates B, Dufek J (2000). Contributions of lower extremity joints to energy dissipation during landings. Med. Sci. Sports Exerc..

[30] Norcross MF (2013). Lower extremity energy absorption and biomechanics during landing, Part I: Sagittal-plane energy absorption analyses. J. Athl. Train..

[31] Norcross MF (2013). Lower extremity energy absorption and biomechanics during landing, Part II: Frontal-plane energy analyses and interplanar relationships. J. Athl. Train..

[32] Ugalde V, Brockman C, Bailowitz Z, Pollard CD (2015). Single leg squat test and its relationship to dynamic knee valgus and injury risk screening. PM&R.

[33] Herrington L, Munro A (2014). A preliminary investigation to establish the criterion validity of a qualitative scoring system of limb alignment during single-leg squat and landing. J. Exerc. Sports Orthop..

[34] Tamura A (2017). Dynamic knee valgus alignment influences impact attenuation in the lower extremity during the deceleration phase of a single-leg landing. PLoS One.

[35] Kadaba MP, Ramakrishnan H, Wootten M (1990). Measurement of lower extremity kinematics during level walking. J. Orthop. Res..

[36] van Melick N, Meddeler BM, Hoogeboom TJ, Nijhuis-van der Sanden MW, van Cingel RE (2017). How to determine leg dominance: The agreement between self-reported and observed performance in healthy adults. PLoS ONE.

[37] Hollman JH, Hohl JM, Kraft JL, Strauss JD, Traver KJ (2012). Effects of hip extensor fatigue on lower extremity kinematics during a jump-landing task in women: A controlled laboratory study. Clin. Biomech. (Bristol, Avon).

[38] Howenstein J, Kipp K, Sabick M (2020). Peak horizontal ground reaction forces and impulse correlate with segmental energy flow in youth baseball pitchers. J. Biomech..

[39] Tamura A, Akasaka K, Otsudo T (2020). Energy absorption strategies in the lower extremities during double-leg landings in knee valgus alignment. Appl. Sci..

[40] McBride JM, Nimphius S (2020). Biological system energy algorithm reflected in sub-system joint work distribution movement strategies: Influence of strength and eccentric loading. Sci. Rep..

[41] Moir GL, Munford SN, Snyder BW, Davis SE (2020). Mechanical differences between adolescents and adults during two landing phases of a drop jump task. J. Strength Cond. Res..

[42] Mersmann F, Charcharis G, Bohm S, Arampatzis A (2017). Muscle and tendon adaptation in adolescence: Elite volleyball athletes compared to untrained boys and girls. Front. Physiol..

[43] Quatman-Yates CC, Myer GD, Ford KR, Hewett TE (2013). A longitudinal evaluation of maturational effects on lower extremity strength in female adolescent athletes. Pediatr. Phys. Ther..

[44] Zeller BL, McCrory JL, Kibler WB, Uhl TL (2003). Differences in kinematics and electromyographic activity between men and women during the single-legged squat. Am. J. Sports Med..

[45] McLean SG, Huang X, van den Bogert AJ (2005). Association between lower extremity posture at contact and peak knee valgus moment during sidestepping: Implications for ACL injury. Clin. Biomech. (Bristol, Avon).

[46] Ford K (2006). A comparison of dynamic coronal plane excursion between matched male and female athletes when performing single leg landings. Clin. Biomech. (Bristol, Avon).

[47] Huston L, Wojtys E (1996). Neuromuscular performance characteristics in elite female athletes. Am. J. Sports Med..

[48] Ford KR, Myer GD, Schmitt LC, Uhl TL, Hewett TE (2011). Preferential quadriceps activation in female athletes with incremental increases in landing intensity. J. Appl. Biomech..

[49] Hollman JH (2009). Relationships between knee valgus, hip-muscle strength, and hip-muscle recruitment during a single-limb step-down. J. Sport Rehabil..

[50] Russell PJ, Croce RV, Swartz EE, Decoster LC (2007). Knee-muscle activation during landings: Developmental and gender comparisons. Med. Sci. Sports Exerc..

[51] Kiapour AM (2015). Uni-directional coupling between tibiofemoral frontal and axial plane rotation supports valgus collapse mechanism of ACL injury. J. Biomech..

[52] Ortiz A (2010). Fatigue effects on knee joint stability during two jump tasks in women. J. Strength Cond. Res. Natl. Strength Cond. Assoc..

[53] Moksnes H, Grindem H (2016). Prevention and rehabilitation of paediatric anterior cruciate ligament injuries. Knee Surg. Sports Traumatol. Arthrosc..

